# Somatic CG6015 mediates cyst stem cell maintenance and germline stem cell differentiation via EGFR signaling in *Drosophila* testes

**DOI:** 10.1038/s41420-021-00452-w

**Published:** 2021-04-06

**Authors:** Qianwen Zheng, Xia Chen, Chen Qiao, Min Wang, Wanyin Chen, Xiaojin Luan, Yidan Yan, Cong Shen, Jie Fang, Xing Hu, Bo Zheng, Yibo Wu, Jun Yu

**Affiliations:** 1 Department of Gynecology, the Affiliated Hospital of Jiangsu University, Jiangsu University, 212001 Zhenjiang, Jiangsu P.R. China; 2 Department of Clinical Pharmacy, the Affiliated Hospital of Jiangsu University, Jiangsu University, 212001 Zhenjiang, Jiangsu P.R. China; 3 State Key Laboratory of Reproductive Medicine, Center for Reproduction and Genetics, Suzhou Municipal Hospital, The Affiliated Suzhou Hospital of Nanjing Medical University, Gusu School, Nanjing Medical University, 215002 Suzhou, Jiangsu P.R. China; 4 Human Reproductive and Genetic Center, Affiliated Hospital of Jiangnan University, 214062 Wuxi, Jiangsu P.R. China

**Keywords:** Cell death, Differentiation

## Abstract

Stem cell niche is regulated by intrinsic and extrinsic factors. In the *Drosophila* testis, cyst stem cells (CySCs) support the differentiation of germline stem cells (GSCs). However, the underlying mechanisms remain unclear. In this study, we found that somatic CG6015 is required for CySC maintenance and GSC differentiation in a *Drosophila* model. Knockdown of *CG6015* in CySCs caused aberrant activation of dpERK in undifferentiated germ cells in the *Drosophila* testis, and disruption of key downstream targets of EGFR signaling (Dsor1 and rl) in CySCs results in a phenotype resembling that of CG6015 knockdown. CG6015, Dsor1, and rl are essential for the survival of *Drosophila* cell line Schneider 2 (S2) cells. Our data showed that somatic CG6015 regulates CySC maintenance and GSC differentiation via EGFR signaling, and inhibits aberrant activation of germline dpERK signals. These findings indicate regulatory mechanisms of stem cell niche homeostasis in the *Drosophila* testis.

## Introduction

Stem cell homeostasis is regulated by its microenvironments or stem cell niches^1^. In *Drosophila*, the testes contain a well-structured microenvironment comprising terminally differentiated somatic cells (hub cells), germline stem cells (GSCs), and somatic cyst stem cells (CySCs), which provides functional signals for the homeostasis of self-renewal and differentiation^2^. Each GSC is tightly enclosed by two CySCs (see refs. ^2,3^). GSCs and CySCs divide asymmetrically to produce two kinds of daughter cells: one remains in contact with the hub and retains self-renewal characteristics, while the other is displaced from the hub and undergoes initial differentiation^4–6^. GSCs produce gonialblasts (GBs), and CySCs give rise to early somatic cyst cells. With the encapsulation of early somatic cyst cells, GBs undergo transit amplification (TA) with four rounds of mitosis and then enter the meiotic stage before terminal differentiation. Fusomes facilitate connections and communication among germ cells, with morphological changes from “dot” to “bifurcation”^7^. During spermatogenesis, autonomous and nonautonomous cell signals are required for germline differentiation. CySCs encapsulate GSCs to establish a tight connection, and exchange signals with GSCs to support germline differentiation^8–10^. Currently, little is known about the interactions of GSCs in the *Drosophila* testis stem cell niche.

Several classical pathways contribute to germline differentiation in the *Drosophila* testis stem cell niche. The Janus kinase-signal transducers and activators of transcription (JAK-STAT) and bone morphogenetic protein (BMP) signaling pathways promote GSC self-renewal by repressing their differentiation^11,12^. Somatic activation of the Hedgehog (Hh) signaling pathway in CySCs regulates the maintenance of CySC characteristics and GSC fate determination^13^. The epidermal growth factor receptor (EGFR) signaling pathway, a highly conserved pathway, is involved in proliferation, differentiation, and several tumorigenic processes^14–18^. The EGFR signaling pathway is specifically activated in somatic cells by EGFR via Spitz (Spi), which is an EGF ligand secreted by germ cells^19^. Ras, one of the best-known downstream targets of EGFR, activates the mitogen-activated protein kinase (MAPK) cascade, which includes Raf kinase, MEK kinase, and ERK kinase. Phosphorylated ERK (dpERK) is recruited into the nucleus and activates downstream transcription factors through phosphorylation modification^20^. The EGFR signaling pathway in CySCs promotes TA division in GSCs in *Drosophila*^19,21^. Aberrations in the special microenvironment created by somatic cells result from a serious loss of EGFR signaling in somatic cells, causing defective encapsulation of GBs and accumulation of early-stage germ cells^14,22^. Higher activation levels of EGFR signaling in somatic cyst cells are necessary for the completion of germline terminal differentiation in *Drosophila* testes^23^. The EGFR signaling pathway drives germline differentiation indirectly by inhibiting BMP signals in *Drosophila* ovaries^24^. Genome-wide RNAi screening of the *Drosophila* S2 cells revealed a vital role of the splicing process in MAPK expression^25^, which could indicate that the EGFR signaling pathway plays a role in regulating somatic lineage.

Some intrinsic factors that control germline differentiation in CySCs have recently been identified^26^. Spliceosome structure and function have been extensively studied; essential spliceosome components like U2A are instrumental in regulating spermatogonial differentiation in *Drosophila*^27^. In addition, heterogeneous nuclear ribonucleoproteins (hnRNPs), heterogeneous nuclear ribonucleoprotein L (hnRNPL), and RNA-binding motif protein, X-linked-like-2 (RBMXL2), are essential for normal spermatogenesis in humans^28,29^. The yeast *Prp17* (or *CDC40)* gene is involved in signaling, cell cycle progression, splicing, and development^30–33^. *CG6015*, the homolog of *Prp17* in *Drosophila*^34^, has been identified as a spliceosome-related gene by a large-scale RNAi screen in *Drosophila* testes and contributes to GSC maintenance and differentiation^35^. However, the regulatory mechanism of CG6015 in the *Drosophila* testis remains unclear.

In this study, the function of CG6015 and the underlying mechanisms were systemically analyzed using in vivo and in vitro approaches in *Drosophila*. Our data showed that somatic CG6015 and Dsor1 are required for CySC maintenance and GSC differentiation. Moreover, knockdown of *rolled* (*rl*), driven by tj-Gal4, causes GSC differentiation defects, but does not affect the maintenance of cyst cells. Surprisingly, somatic silencing of *CG6015*, *Dsor1*, and *rl* results in the ectopic expression of dpERK in undifferentiated germ cells (germline dpERK signals) in *Drosophila* testes. Our data suggest a novel mechanism involving CG6015 and EGFR signaling, which modulates the differentiation process in the *Drosophila* testis stem cell niche.

## Results

### Somatic CG6015 is required for CySC maintenance and GSC differentiation

**Fig. 1:**
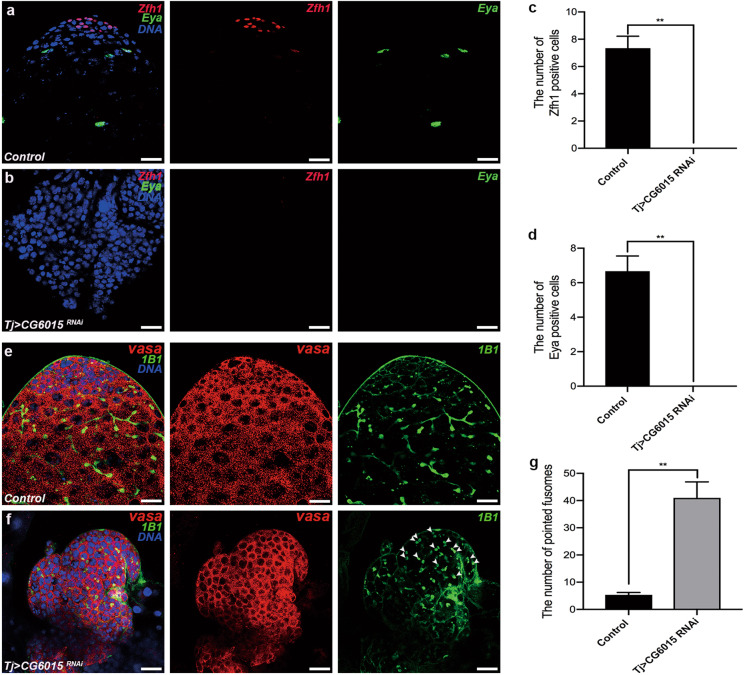
The necessity of somatic CG6015 for CySC maintenance and GSC differentiation in *Drosophila* testes. **a**, **b** Immunostaining of control (**a**) and *tj* > *CG6015 RNAi* (**b**) testes for Zfh1 (red) and Eya (green). **c** Number of Zfh1-positive cells in control (*n* = 3) and *tj* > *CG6015 RNAi* (n = 3) testes. **d** Number of Eya-positive cells in control (*n* = 3) and *tj* > *CG6015 RNAi* (*n* = 3) testes. **e**, **f** Immunostaining of control (**e**) and *tj* > *CG6015 RNAi* (**f**) testes for Vasa (red) and 1B1 (green). **g** Number of pointed fusomes in control (*n* = 3) and *tj* > *CG6015 RNAi* (*n* = 3) testes. The W^1118^ line was used as control. Student’s *t* test was used for the statistical analysis. **P < 0.01. Scale bar: 20 μm.

To determine the functions of somatic CG6015 in *Drosophila* testes, an RNAi-mediated analysis was conducted, driven by tj-GAL4. Zinc-finger homeodomain protein 1 (Zfh1) is a transcription factor that is highly expressed in CySCs and somatic cyst cells surrounding the hub^36^. High levels of eyes absent (Eya), a mature cyst cell marker, are typically observed in late-stage somatic cyst cells^37^. Unexpectedly, Zfh1 and Eya have not been detected in *tj* > *CG6015 RNAi* testes, indicating total loss of CySCs and mature cyst cells (Fig. 1a–d).

Vasa was used as a germ cell marker in the testis^38^, and fusomes were labeled with 1B1 to observe the differentiation process^39^. Control testes displayed a strong pattern with the formation of pointed and branched fusomes in Vasa-positive germ cells (Fig. 1e). However, accumulation of undifferentiated germ cells and pointed fusomes was observed in *tj* > *CG6015 RNAi* testes (Fig. 1f). The number of pointed fusomes in *tj* > *CG6015 RNAi* testes was significantly higher than that in controls (Fig. 1g). These results suggested that CG6015 regulates CySC characteristics, and nonautonomously affects germ cell differentiation.

### Inactivation of somatic CG6015 causes differentiation defects without hub signals and modulates germ cell apoptosis and proliferation

**Fig. 2:**
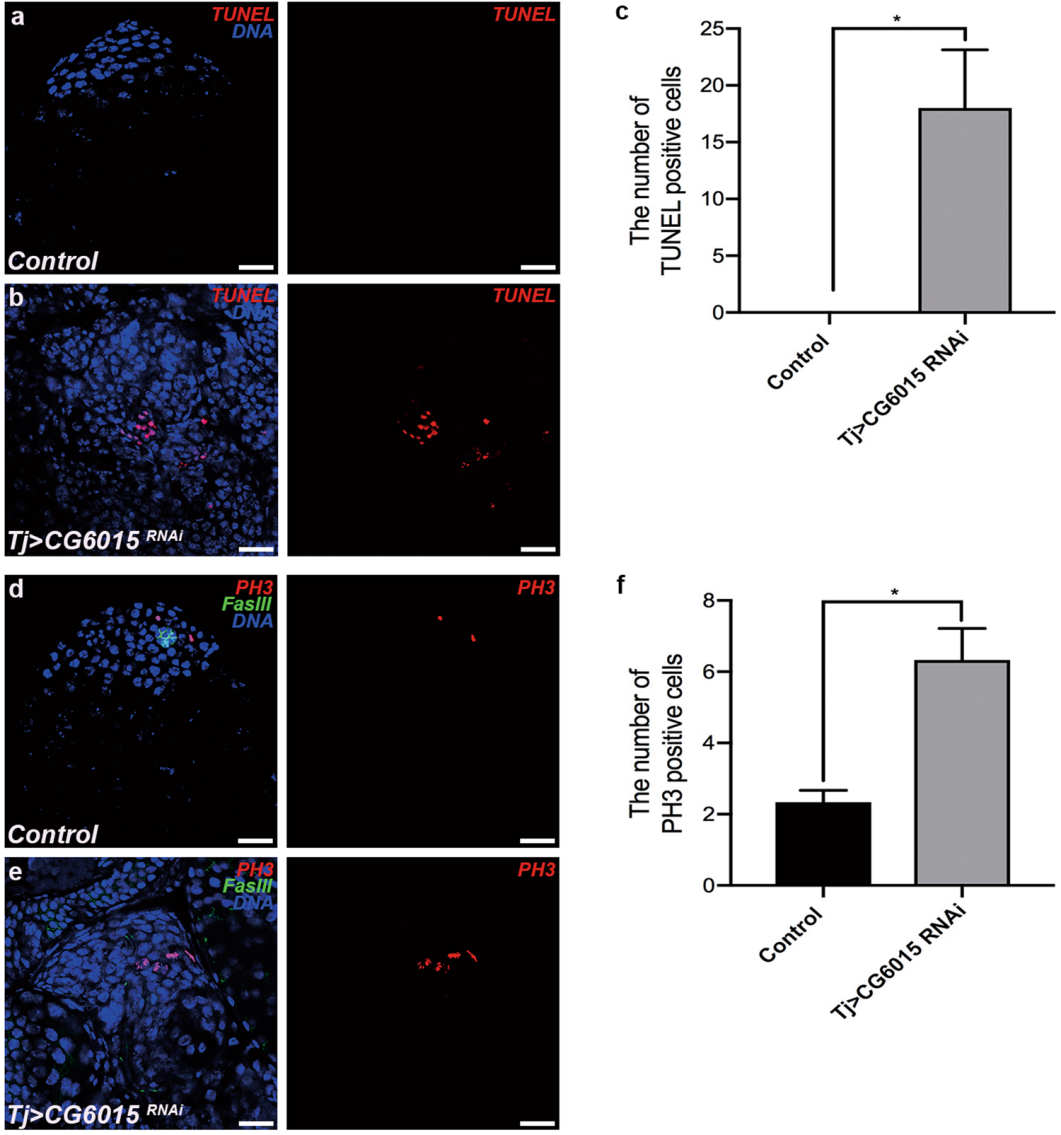
Knockdown of *CG6015* in CySCs leads to cell death and proliferation dysfunction. **a**, **b** TUNEL (red) staining of control (**a**) and *tj* > *CG6015 RNAi* (**b**) testes. **c** Number of TUNEL-positive cells in control (*n* = 3) and *tj* > *CG6015 RNAi* (*n* = 3) testes. **d**, **e** Immunostaining of control (**d**) and *tj* > *CG6015 RNAi* (**e**) testes for PH3 (red) and FasIII (green). **f** Number of PH3-positive cells in control (*n* = 3) and *tj* > *CG6015 RNAi* (*n* = 3) testes. The W^1118^ line was used as control. Student’s *t* test was used for the statistical analysis. *P < 0.05. Scale bar: 20 μm.

To characterize the accumulated undifferentiated germ cell cysts, we detected apoptosis and proliferation in the *Drosophila* testis. TUNEL-positive cells dramatically increased in *tj* > *CG6015 RNAi* testes (Fig. 2a–c). Fasciclin III (FasIII) protein and phosphohistone-3 (PH3) are markers of hub cells and M-phase cells, respectively. Surprisingly, no hub cells were observed around the accumulated undifferentiated germ cell cysts, while PH3-positive cells were significantly higher in *tj* > *CG6015 RNAi* testes than in the controls (Fig. 2d–f). These results suggested that apoptosis and proliferation were maintained without hub signals in undifferentiated germ cells induced by somatic CG6015 inactivation.

### CG6015 regulates proliferation and apoptosis in S2 cells

**Fig. 3:**
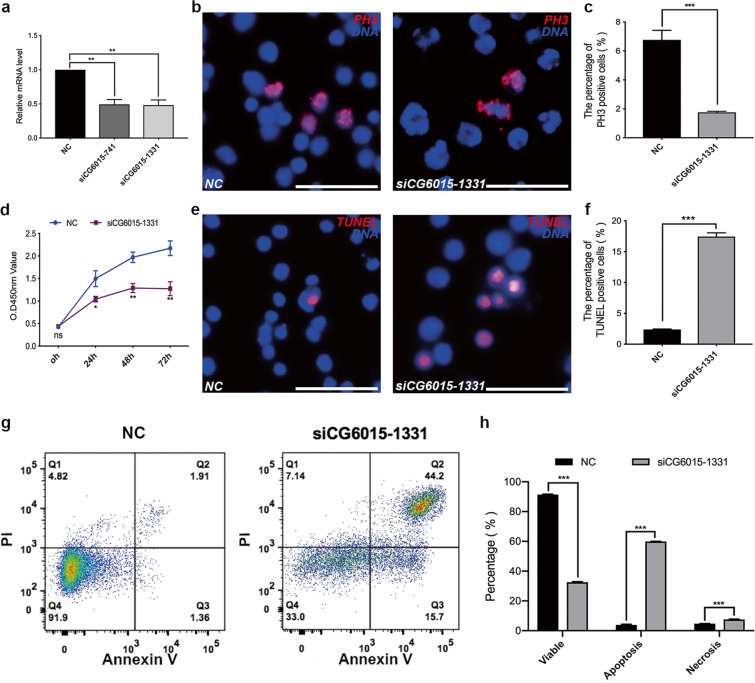
Functional analysis of CG6015 in S2 cells. **a** Relative mRNA levels of *CG6015* in the negative control (NC) and *siCG6015* (*siCG6015-741* and *siCG6015-1331*)-treated S2 cells to validate knockdown efficiency. **b** Immunostaining of NC and *siCG6015-1331*-treated S2 cells by using PH3 (red). **c** Percentages of PH3-positive cells in NC (*n* = 3) and *siCG6015-1331*-treated (*n* = 3) S2 cells. **d** CCK-8 test in NC and *siCG6015-1331*-treated S2 cells. **e** TUNEL (red) staining in NC and *siCG6015-1331*-treated S2 cells. **f** Percentages of TUNEL-positive cells in NC (*n* = 3) and *siCG6015-1331*-treated (*n* = 3) S2 cells. **g** Cell component analysis in NC and *siCG6015-1331*-treated S2 cells by flow cytometry. **h** Percentages of cell components in NC (*n* = 3) and *siCG6015-1331*-treated (*n* = 3) S2 cells. The Student’s *t* test was used for the statistical analysis. *P < 0.05; **P < 0.01; ***P < 0.001; n.s. not significant. Scale bar: 30 μm.

The function CG6015 was further analyzed in vitro. Two small-interfering RNAs (siRNAs: *siCG6015-741* and *siCG6015-1331*) were used to silence the mRNA expression of *CG6015*, and *siCG6015-1331* was selected for further functional analysis in S2 cells (Fig. 3a). First, we found that the relative mRNA expression levels of Prp complex subunits (*Prp19* and *Prp18*) and Sm complex subunits (*SmB, SmD1*, and *SmF*) were upregulated after silencing of *CG6015* (Supplementary Fig. S1). We also observed that *siCG6015* decreased the proportion of PH3-positive S2 cells (Fig. 3b, c). CCK-8 assay was simultaneously conducted to assess cell growth conditions after silencing of *CG6015*, and the results showed clear suppression of cell growth ability (Fig. 3d). In addition, apoptosis was detected using the TUNEL assay and flow cytometry. Interestingly, the ratio of TUNEL-positive cells increased after *CG6015* knockdown (Fig. 3e, f), indicating that *siCG6015* treatment promoted apoptosis. Flow cytometric cell component tests confirmed these results and showed that the ratio of apoptosis and necrosis significantly increased in S2 cells after treatment with *CG6015* siRNA (Fig. 3g, h). These results indicated that CG6015 is essential for cell proliferation and cell death.

### Somatic CG6015 inactivation disrupts the expression pattern of dpERK

**Fig. 4:**
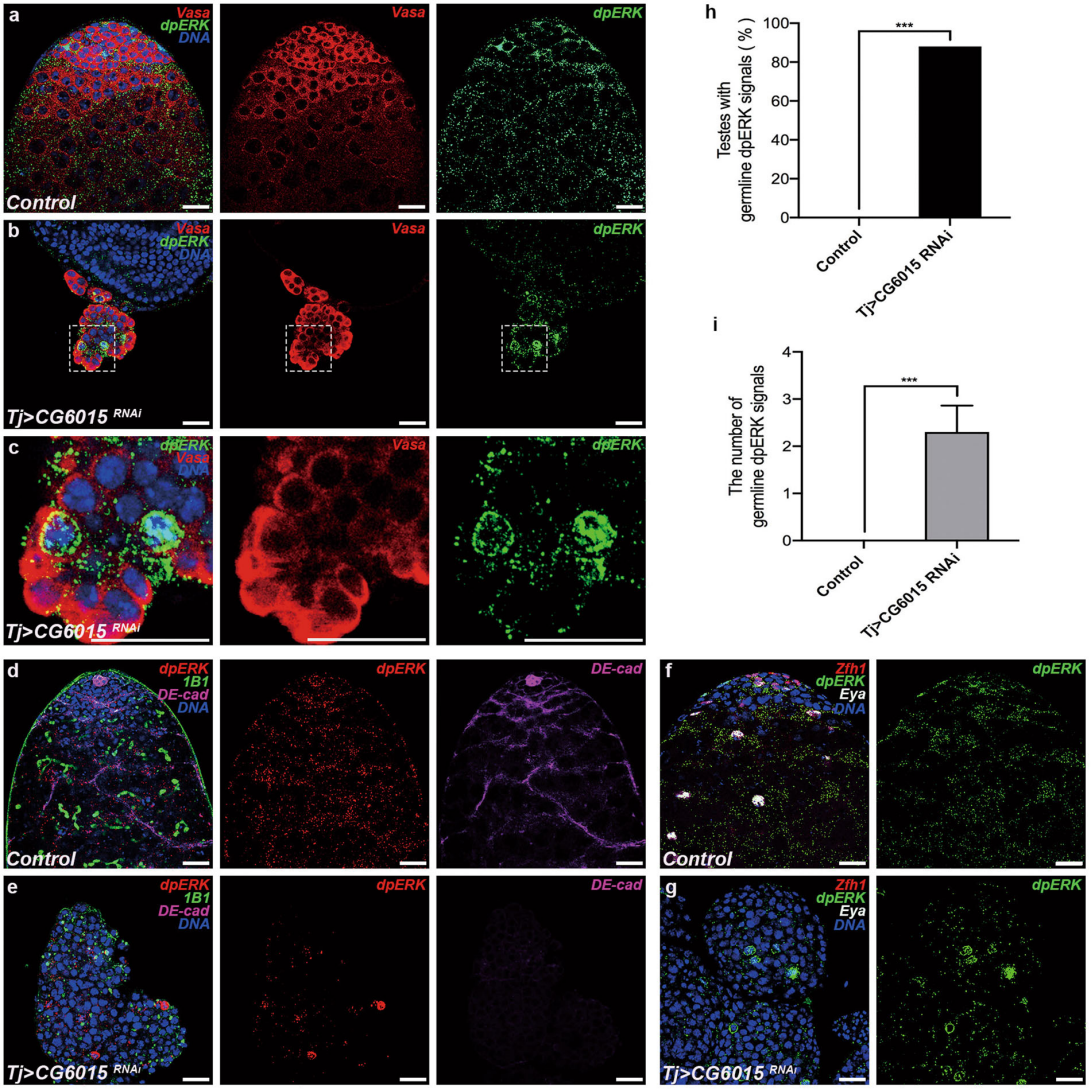
Somatic CG6015 inactivation leads to aberrant dpERK expression. **a**–**c** Immunostaining of control (**a**) and *tj* > *CG6015 RNAi* (**b**, **c**) testes for Vasa (red) and dpERK (green). Panel (**c**) shows enlarged views of sections marked in (**b**). **d**, **e** Immunostaining of control (**d**) and *tj* > *CG6015 RNAi* (**e**) testes for dpERK (r**e**d), 1B1 (green), and DE-cad (magenta). **f**, **g** Immunostaining of control (**f**) and *tj* > *CG6015 RNAi* (**g**) testes for Zfh1 (red), dpERK (green), and Eya (gray). **h** Percentages of testes with germline dpERK signals in control (*n* = 20) and *tj* > *CG6015 RNAi* (*n* = 16) testes. Chi-square test was used to evaluate for statistical differences of testes with germline dpERK signals. **i** Number of germline dpERK signals in control (*n* = 10) and *tj* > *CG6015 RNAi* (*n* = 10) testes. The Student’s *t* test was used for statistical differences of the number of germline dpERK signals. ***P < 0.001. The W^1118^ line was used as control. Scale bar: 20 μm.

EGFR signaling in somatic cells has critical functions in GSC differentiation^22,40^. We aimed to determine whether CG6015 mediates GSC differentiation via EGFR signaling by detecting dpERK, a key downstream activator of the EGFR signaling pathway^41^. In the control testes, dpERK was mainly detected in somatic cyst cells (Fig. 4a), as described previously^42^. Conversely, we observed ectopic dpERK signals, mostly mislocalized in germ cell nuclei, in accumulated undifferentiated germ cell cysts after knockdown of *CG6015* driven by tj-GAL4 (Fig. 4b, c). Moreover, the mislocalized germline dpERK signals were accompanied by pointed fusomes (Fig. 4d, e), and were not co-located with Zfh1 or Eya (Fig. 4f, g). Importantly, the percentage of testes with mislocalized germline dpERK signals increased to 88% (Fig. 4h). Further, the number of mislocalized germline dpERK signals increased by an average of 2.30 ± 0.56 in *tj* > *CG6015 RNAi* testes (Fig. 4i). Thus, inactivation of *CG6015* in somatic cyst cells led to aberrant activation of ERK kinase in germ cells, along with differentiation defects, suggesting a possible role for CG6015 and EGFR signaling in germline differentiation in the stem cell niche.

### Dsor1 is required for CySC maintenance and GSC differentiation in CySC lineages

**Fig. 5:**
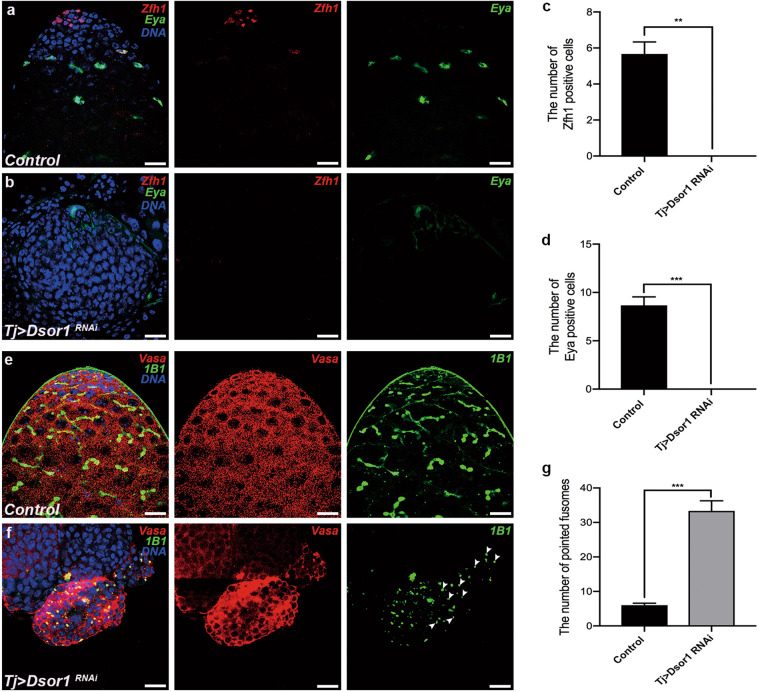
Somatic Dsor1 is indispensable for CySC maintenance and GSC differentiation. **a**, **b** Immunostaining of control (**a**) and *tj* > *Dsor1 RNAi* (**b**) testes for Zfh1 (red) and Eya (green). **c** Number of Zfh1-positive cells in control (n = 3) and *tj* > *Dsor1 RNAi* (*n* = 3) testes. **d** Number of Eya-positive cells in control (*n* = 3) and *tj* > *Dsor1 RNAi* (n = 3) testes. **e**, **f** Immunostaining of control (**e**) and *tj* > *Dsor1 RNAi* (**f**) testes for Vasa (red) and 1B1 (green). **g** Number of pointed fusomes in control (*n* = 3) and *tj* > *Dsor1 RNAi* (*n* = 3) testes. The W^1118^ line was used as control. The Student’s *t* test was used for the statistical analysis. **P < 0.01; ***P < 0.001. Scale bar: 20 μm.

MEK, also known as downstream of raf (Dsor1), is the direct phosphorylation activator of ERK (also called rl in *Drosophila*)^43^. To determine the exact roles of Dsor1 and elucidate the relationship between CG6015 and EGFR signaling in CySC lineages, we knocked down *Dsor1* driven by tj-Gal4. Neither Zfh1 nor Eya signal was detected after knocking down of *Dsor1* in CySCs (Fig. 5a–d), indicating that Dsor1 is essential for CySC maintenance. Further, undifferentiated germ cells accumulated and pointed fusomes increased in *tj* > *Dsor1 RNAi* testes (Fig. 5e–g). Higher TUNEL signals were observed in *tj* > *Dsor1 RNAi* testes when compared with control testes (Supplementary Fig. S2a–c). In addition, undifferentiated germ cells in *tj* > *Dsor1 RNAi* testes could proliferate without hub signals (Supplementary Fig. S2d–f). These results suggested that Dsor1 could phenocopy CG6015 in *Drosophila* testes.

### Somatic downregulation of rl disrupts GSC differentiation

rl is a key downstream target of the EGFR signaling pathway^43^. We observed Zfh1-positive cells and Eya-positive cells in *tj* > *rl RNAi* testes (Supplementary Fig. S3a, b), indicating that rl was not necessary for the maintenance of CySCs. No significant difference was observed in the quantity of Zfh1-positive cells, while the number of Eya-positive cells significantly decreased (Supplementary Fig. S3c, d). Unlike in the control testes, early germ cells dramatically accumulated, with increased pointed fusomes, in the *tj* > *rl RNAi* testes (Supplementary Fig. S3e–g). Although hub cells existed in all testes, the number of TUNEL-positive and PH3-positive cells increased in *tj* > *rl RNAi* testes (Supplementary Fig. S4). These results emphasized the crucial roles of rl in the somatic cyst lineage, in the promotion of GSC differentiation.

### EGFR signaling regulates the survival of Drosophila S2 cells

**Fig. 6:**
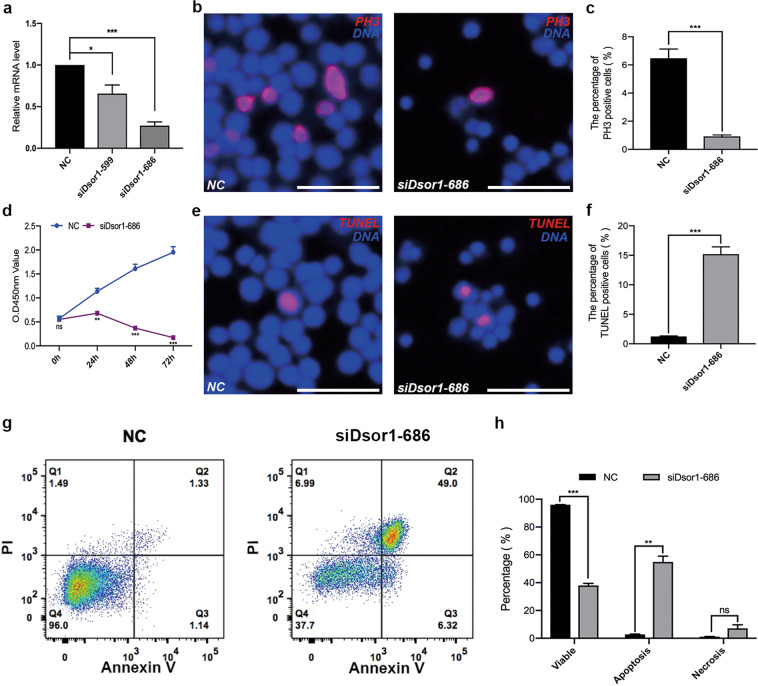
Dsor1 regulates proliferation and apoptosis in S2 cells. **a** Relative mRNA levels of *Dsor1* in the negative control (NC) and *siDsor1* (*siDsor1-599* and *siDsor1-686*)-treated S2 cells to validate knockdown efficiency. **b** Immunostaining of PH3 (red) in NC and *siDsor1-686*-treated S2 cells. **c** Proportions of PH3-positive cells in NC (*n* = 3) and *siDsor1-686*-treated (*n* = 3) S2 cells. **d** CCK-8 test in NC and *siDsor1-686*-treated S2 cells. **e** TUNEL (red) staining in NC and *siDsor1-686*-treated S2 cells. **f** Percentages of TUNEL-positive cells in NC (*n* = 4) and *siDsor1-686*-treated (*n* = 4) S2 cells. **g** Flow cytometry test for cell components in NC and *siDsor1-686*-treated S2 cells. **h** Percentages of cell components in NC (*n* = 3) and *siDsor1-686*-treated (*n* = 3) S2 cells. The Student’s *t* test was used for the statistical analysis. *P < 0.05; **P < 0.01; ***P < 0.001; n.s. not significant. Scale bar: 30 μm.

To elucidate the function of the EGFR signaling pathway, we used multiple small-interfering RNAs (*siDsor1-599* and *siDsor1-686* and *sirl-51* and *sirl-785*) to downregulate the expression of *Dsor1* and *rl*, respectively, in S2 cells. *siDsor1-686* and *sirl-785*, selected using qRT-PCR, were used for the functional analysis (Fig. 6a and Supplementary Fig. S5a). We found that the percentage of PH3-positive signals was dramatically lower in the *siDsor1-686* and *sirl-785* groups than in the control groups (Fig. 6b, c and Supplementary Fig. S5b, c). Further, apparent inhibition of cell growth was observed using CCK-8 assay after silencing *Dsor1* or *rl* (Fig. 6d and Supplementary Fig. S5d), suggesting that blockage of EGFR signaling pathway might reduce cell survival. Moreover, to determine whether Dsor1 and rl were involved in apoptosis, TUNEL and flow cytometry assays were performed separately. The percentage of TUNEL-positive cells (Fig. 6e, f and Supplementary Fig. S5e, f), and the ratio of apoptotic cells dramatically increased after silencing *Dsor1* or *rl*, compared with control (Fig. 6g, h and Supplementary Fig. S5g, h). Thus, our results indicated that Dsor1 and rl, as key downstream targets of EGFR signaling, are essential for S2 cell survival.

### Inactivation of Dsor1 and rl in CySCs disrupts the expression pattern of dpERK

**Fig. 7:**
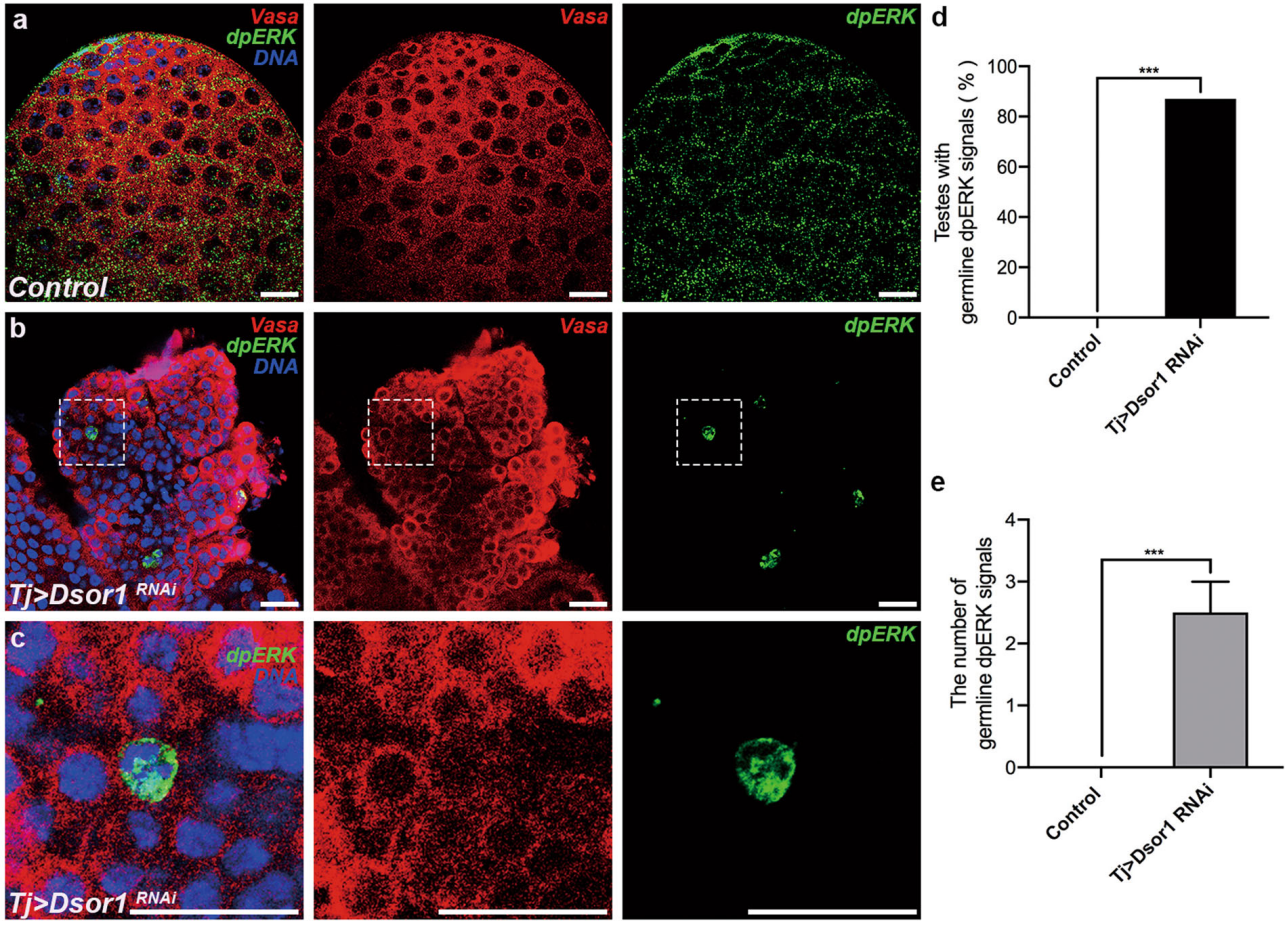
Somatic Dsor1 inactivation causes ectopic dpERK expression in undifferentiated germ cells. **a**–**c** Immunostaining of Vasa (red) and dpERK (green) in control (**a**) and *tj* > *Dsor1 RNAi* (**b**, **c**) testes. Images in (**c**) are enlarged views of the areas marked in (**b**). **d** Percentages of testes with germline dpERK signals in control (*n* = 20) and *tj* > *Dsor1 RNAi* (*n* = 15) testes. Chi-square test was used to evaluate for statistical differences of testes with germline dpERK signals. **e** The number of germline dpERK signals in control (*n* = 10) and *tj* > *Dsor1 RNAi* (*n* = 10) testes. The Student’s *t* test was used for statistical differences of the number of germline dpERK signals. ***P < 0.001. The W^1118^ line was used as control. Scale bar: 20 μm.

Since our results revealed a role for CG6015 in GSC differentiation via EGFR signaling, and somatic CG6015 could cause mislocalized expression of dpERK in germ cells, we then determined whether germline dpERK signals reappeared in accumulated undifferentiated germ cells after the downregulation of *Dsor1* and *rl*. Surprisingly, a significant accumulation of aberrantly expressed dpERK signals was detected in the undifferentiated germ cells, compared with control testes (Fig. 7a–c and Supplementary Fig. S6a–c). The percentage of testes with mislocalized germline dpERK signals increased in *tj* > *Dsor1 RNAi* testes (87%, Fig. 7d) and *tj* > *rl RNAi* testes (78%, Supplementary Fig. S6d). Further, the number of mislocalized germline dpERK signals increased by an average of 2.50 ± 0.50 in *tj* > *Dsor1 RNAi* testes (Fig. 7e) and 3.22 ± 0.95 in *tj* > *rl RNAi* testes (Supplementary Fig. S6e). The inactivation of the EGFR signaling pathway led to germline differentiation defects and activated ectopic expression of dpERK, providing novel evidence of EGFR-mediated differentiation defects and ERK kinase activation in germ cells.

## Discussion

In this study, we determined the functions of somatic CG6015 in CySC maintenance and GSC differentiation and elucidated novel mechanisms involving CG6015 and EGFR signaling. We discovered that CG6015 and key targets of the EGFR signaling pathway (Dsor1 and rl) were present in CySCs and mediated GSC differentiation by non-cell-autonomous effects. Importantly, germline dpERK signals were activated and observed among the accumulated undifferentiated germ cells in the *Drosophila* testis after the disruption of CG6015, Dsor1, and rl in CySCs.

**Fig. 8:**
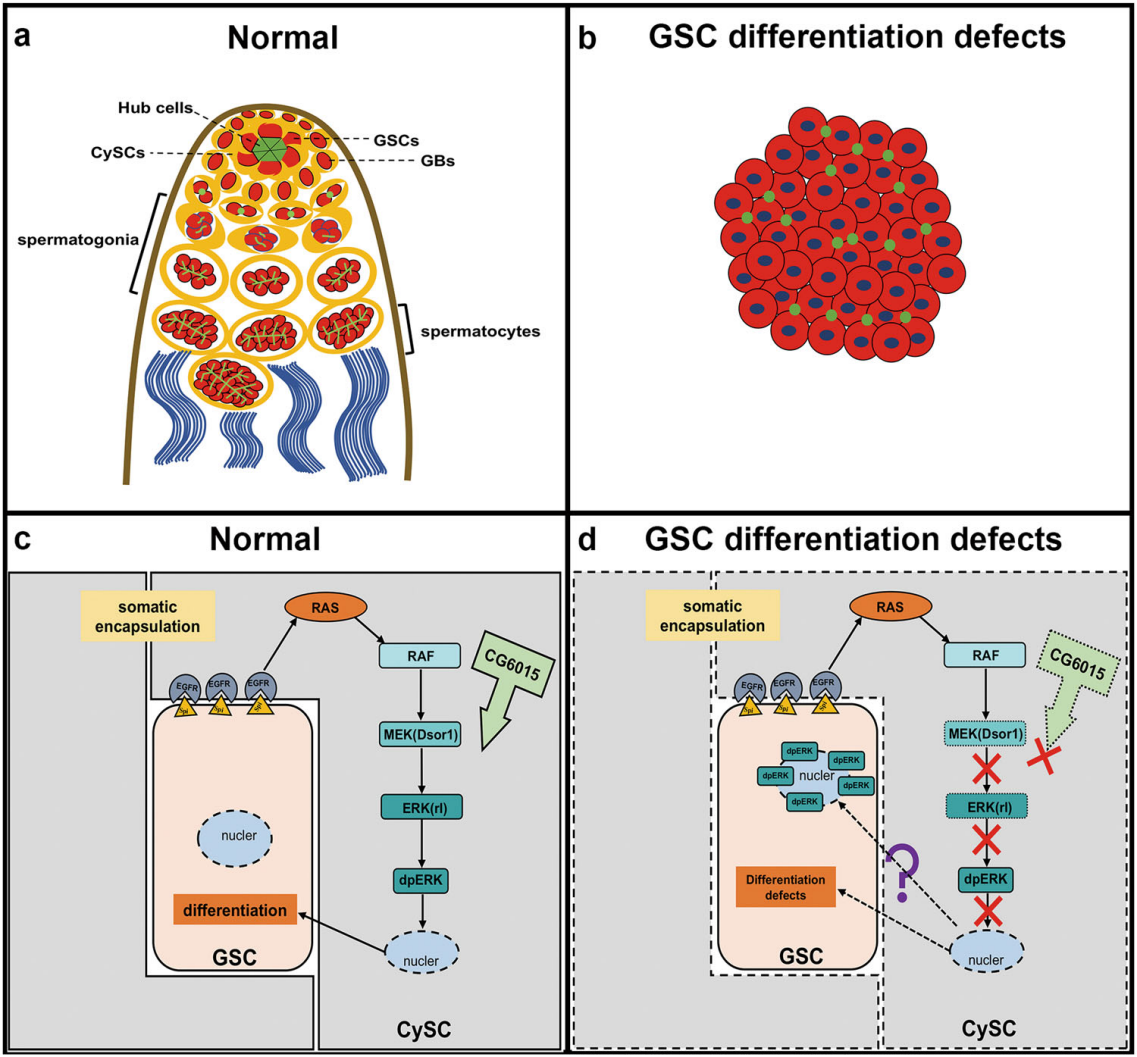
Schematic representation of CG6015 and EGFR signaling in the *Drosophila* testis. **a**, **b** Graphical representation of the apical tips of normal testes and testes with GSC differentiation defects. **c**, **d** Mechanisms of CG6015 and EGFR signaling. In normal testes, the stem cell niche is composed of GSCs, CySCs, and hub cells, which occupy the apical tips of the testis. For the integrity of germ cell differentiation, somatic cells should envelop germ cells, and fusomes gradually transition from dot to bifurcate. Somatic inactivation of CG6015 and key targets in the EGFR signaling pathway lead to the accumulation of undifferentiated germ cells, and eventually the formation of tumor-like germ cell cysts with pointed fusomes in the *Drosophila* testis. Our results show the maintenance of stem cell niche homeostasis by CG6015 and EGFR signaling via germline dpERK signals.

Preliminary evidence identified CG6015 as a GSC regulator in the *Drosophila* testis^35^. Here, we found that inactivation of CG6015 in CySCs led to GSC differentiation defects, and promoted self-renewal and apoptosis of the accumulated undifferentiated germ cells without normal niche signals. Somatic CG6015, which induced GSC differentiation defects, unexpectedly activated the aberrant expression of germline dpERK signals in undifferentiated germ cells. Importantly, Dsor1 or rl mimicked the phenotype of CG6015 in *Drosophila* testes and S2 cells, indicating possible correlations between CG6015 and EGFR signaling pathway (Fig. 8).

Somatic cells are required to encapsulate germ cells for differentiation^14^. Several studies have demonstrated the roles of EGFR signaling in germline differentiation via EGFR, Ras, and Raf factors^23,26,42,44^. However, the role of Dsor1 and rl in the homeostasis of the stem cell niche in the *Drosophila* testis was not extensively studied. Dsor1 is a downstream target that could directly phosphorylate ERK protein^45^. The mRNA expression of *rl* could be affected by some splicing factors^25^. Our results also revealed that one such splicing factor, *CG6015*, mediated expression changes of several spliceosome components, indicating regulatory roles for CG6015 in spliceosomes. We, therefore, hypothesized that CG6015 likely plays a critical role in mRNA splicing, and regulates EGFR signaling for germ cell differentiation. Inactivation of CG6015 and its targets in EGFR signaling in CySCs disrupted the balance of stem cell niche signals, leading to failure of GSC differentiation.

Considering the functions of Dsor1 and rl in the testis, they likely act via more pathways than only the MEK–ERK cascade to control somatic behavior. EGFR could also activate Vav/Rac1/Rho1 signals to display similar germ cell enclosure functions. Rho1, a negative regulatory factor of rl, could regulate germ cell enclosure antagonistically against EGFR/Vav/Rac1 (see ref. ^19^). Rho1 is a small GTPase, which regulates the actin cytoskeleton. Several signaling pathways modulate cell motility and cell shape by controlling cytoskeleton dynamics^46^. Importantly, a somatic permeability barrier has been discovered to regulate germline encapsulation and differentiation^47^. Hence, Rho1-mediated cytoskeleton changes could damage germline encapsulation by affecting the somatic permeability barrier. Further studies are needed to elucidate the detailed mechanisms by which EGFR signaling modulates GSC encapsulation and differentiation.

Normally, dpERK is activated and expressed in CySCs and somatic cyst cells of the testis^23^. The initial discovery of ectopically expressed dpERK signals in undifferentiated germ cells was unexpected. To confirm the localization of these ectopic dpERK signals, co-staining of Vasa and dpERK was performed. The possibility of co-localization between the ectopic dpERK signals and somatic lineages was assessed and excluded. Germline dpERK signals were reported for the first time in our study. We speculated whether GSC differentiation defects induced the activation of germline dpERK signals, providing us with a specific understanding of the germline EGFR signaling pathway. The effects of MAPK cascades in cell-fate determination, such as proliferation, apoptosis, and differentiation, have been extensively investigated^48^. The localization of dpERK in the nucleus or cytoplasm was regarded as a switch to determine proliferation and differentiation in mouse muscle progenitors. Increased dpERK nuclear translocation could repress myogenic differentiation in head muscle progenitors^49^. Prevention of ERK nuclear translocation has been studied as a novel therapeutic strategy for some Ras/ERK-related cancers^50^. This is a possible explanation for the nuclear accumulation of the germline dpERK signals. However, the detailed mechanism requires further study.

In summary, we discovered that CG6015 was required in CySCs for GSC differentiation through EGFR signaling. Our discovery of activated germline dpERK signals could greatly improve our understanding of stem cell niche regulation, male fertility, and germline tumorigenesis.

## Materials and methods

### Fly stocks and fly crosses

All flies were raised on standard cornmeal molasses agar medium at 25 °C. The transgenic RNA interference (RNAi) flies used in this study were obtained from TsingHua Fly Center (THFC) and were derived from the same RNAi collection as the Transgenic RNAi Project. UAS-RNAi flies included *UAS-CG6015 RNAi* (THU1409), *UAS-Dsro1 RNAi* (THU0677), and *UAS-rl RNAi* (THU3530). Tj-Gal4 (104055) was acquired from *Drosophila* Genetic Resource Consortium (DGRC). The W^1118^ line was used as control.

UAS/Gal4 system was used to mediate tissue-specific expressed knockdown in *Drosophila*. Two-to-three-day-old flies were used in this study. Males from tj-Gal4 line were randomly selected to cross with transgenic UAS-RNAi virgin females and raised at 25 °C. Then we chose qualified male offsprings with specific genotypes for further functional analysis.

### Cell culture and transfection

*Drosophila* S2 cells were obtained from *Drosophila* Genomics Resource Center and were cultured in Schneider’s *Drosophila* medium (21720024, Gibco, USA) supplemented with 10% heat-inactivated fetal bovine serum (FBS) (04-001-1ACS, Bioind, Israel) at 28 °C. Every 3–4 days, S2 cells were passaged to another plate in 1:3 or 1:2 ratio as previously described^51^. Cells have been tested and confirmed to be free of mycoplasma contamination.

Before transfection, S2 cells were seeded into a six-well plate to guarantee that the cell growth area reached 70–80% of the well. For knocking down target genes, Lipofectamine 2000 Transfection Reagent (Lipo2000, 11668019, Invitrogen, USA), Opti-Minimal Essential Medium (MEM) (31985-062, Gibco, USA), and siRNA were used together. The detailed transfected process was as follows: two tubes were prepared to mix reagent, the first one contained 250 μl Opti-MEM and 15 μl Lipo2000 and incubated for 5 min after vortexing for 5 s at room temperature. Then, the other one contained 250 μl Opti-MEM, and 15 μl siRNA was mixed with the first tube and incubated for 20 min after vortexing for 5 s at room temperature. The siRNAs were designed and synthesized by GenePharma (Suzhou, China). The detailed information of siRNAs is listed in Supplementary Table S1.

### Quantitative real-time PCR

The total RNA was extracted from S2 cells using RNAiso Plus kit (9108, Takara, Japan). cDNA was synthesized by Prime Script RT Reagent Kit (RR037A, Takara, Japan). TB Green^TM^ Premix Ex Taq^TM^ (RR420A, Takara, Japan) was used to carry out qRT-PCR and GAPDH was amplified as an internal standard. Fold changes were calculated using the standard curve according to the manufacturer’s protocol. Each experiment was repeated three times independently. Information of all primers used for qRT-PCR is listed in Supplementary Table S2.

### Immunostaining and antibodies

Fly testes were dissected in 1× PBS and fixed for 30 min in 4% PFA. They were washed three times in 0.3% PBST and blocked for 30 min in 5% BSA. Testes were incubated with primary antibody overnight at 4 °C. Then the samples were washed three times for 30 min in 0.3% PBST and incubated with secondary antibodies at room temperature for 1 h avoiding light. Testes were then washed three times again by 0.3% PBST. Finally, testes were stained with Hoechst-33342 (1.0 mg/mL, C0031, Solarbio, Beijing, China) for 5 min before finalizing^51^. The images were captured by LSM800 Zeiss confocal microscope and processed by Adobe Photoshop Software. *Drosophila* S2 cells were cultured on cover glasses for 48 h, and immunostaining was performed in 24-well plates according to the similar protocols described above.

The antibodies used in this study included rat anti-Zfh1 (a gift from Tong lab, 1:1000), mouse anti-Eya (#AB_528232, DSHB: Developmental Studies Hybridoma Bank, 1:30), rat anti-Vasa (#AB_760351, DSHB, 1:20), mouse anti-1B1 (#AB_528070, DSHB, 1:50), rabbit anti-PH3 (#53348, CST: Cell Signaling Technology, 1:1000), mouse anti-FasIII (#AB_528238, DSHB, 1:50), rat anti-DE-cadherin (#AB_528120, DSHB, 1:15), and rabbit anti-dpERK (#4370, CST, 1:200). Secondary antibodies containing Cy3, A488, and A647 (Molecular Probes and Jackson Immunological) were diluted at 1:400 with 5% BSA.

### TUNEL assay

Cell death tests in testes and S2 cells were examined by TUNEL BrightRed Apoptosis Detection Kit (A113, Vazyme, Nanjing, China) according to the manufacturer’s protocols. After blocking, testes or S2 cells were incubated in 50 μl of 1× equilibration buffer that was diluted by ddH_2_O at 1:5 (room temperature) for 30 min in the dark. Then, a mixture with 34 μl ddH_2_O, 10 μl 5× equilibration buffer, 5 μl BrightRed Labeling Mix, and 1 μl Recombinant TdT Enzyme was prepared in the dark. After balance, 50 μl mixture was added and incubated with testes or S2 cells for 1 h at 37 °C in the dark. After TUNEL staining, both testes and S2 cells were washed in 1× PBS three times. Before finalizing, testes and S2 cells were stained with Hoechst-33342 (1.0 mg/mL, C0031, Solarbio, Beijing, China) for 5 min.

### Flow cytometry assay

After transfection for 48 h in S2 cells, flow cytometry was conducted by Annexin V-Alexa Fluor 647/propidium iodide (PI) Apoptosis Assay Kit (FMSAV647-100, FcMACS, Nanjing, China). According to the manufacturer’s protocols, S2 cells were washed with ice-cold 1× PBS and resuspended by binding buffer that was diluted with DEPC-treated water at the ratio of 1:4 after centrifugation. In all, 5 μl Annexin V-Alexa Fluor 647 and 10 μl PI were added into 100-μl cell suspension. In total, 1 × 10^6^ cells are required in each sample and incubated for 15 min at room temperature in the dark. Before testing on FACScan flow cytometry (BD Biosciences intervals, San Jose, CA, USA), 200 μl 1× PBS was added into each sample to dilute cells. The experiments required more than three replicates, and the final results were analyzed and processed by FlowJo software.

### Cell viability assay

CCK-8 Cell Counting Kit (A311-01-AA, Vazyme, Nanjing, Chania) was used to analyze cell growth situation in S2 cells. Based on the manufacturer’s instructions, after transfection at 0, 24, 48, and 72 h, S2 cells were collected and resuspended after centrifugation using 10% CCK-8 mixture, which was diluted by Schneider’s *Drosophila* Medium. The cell suspension was seeded into 96-well plates with three accessory wells for each sample and incubated at 37 °C for 2 h. The absorbance in each well was evaluated on a spectrophotometer (Multiskan GO, Thermo Scientific, Waltham, USA) at 450 nm. The results were performed for more than three independent experiments.

### Statistical analysis

All the experiments conducted in this study were repeated at least three times. The quantitative results were presented as means ± standard error of the mean (SEM) and evaluated for statistical differences using Student’s *t* test and one-way ANOVA by Graphpad Software (https://www.graphpad.com/). Chi-square test was used to evaluate for statistical differences of testes with germline dpERK signals. *P < 0.05; **P < 0.01; ***P < 0.001.

## Supplementary information

Supplementary Figure Legends

Supplementary Figure S1

Supplementary Figure S2

Supplementary Figure S3

Supplementary Figure S4

Supplementary Figure S5

Supplementary Figure S6

Supplementary Table S1

Supplementary Table S2
