# Supplementary material for: Somatic CG6015 mediates cyst stem cell maintenance and germline stem cell differentiation via EGFR signaling in Drosophila testes
Source: Cell Death Discov. 2021 Apr 6;7:68. doi: 10.1038/s41420-021-00452-w (PMC8024382; doi:10.1038/s41420-021-00452-w)
Supplement: Supplementary file 1 — Supplementary Figure Legends [file 41420_2021_452_MOESM1_ESM.docx]

**Supplementary Figure Legends**

**Fig. S1 Relative mRNA expression of splicing factors in *Drosophila* S2 cells.** Relative mRNA level of spliceosome subunits (*Prp19*, *Prp18*, *SmB*, *SmD1*, *SmE* and *SmF*) in control and *siCG6015-1331* group of S2 cells. Student’s *t*-test was used for the statistical analysis. *P<0.05; **P<0.01, n.s. not significant. Error bars represent SEM.

**Fig. S2** **Knockdown of *Dsor1* in CySCs increases cell death and proliferation ability in *Drosophila* testes. a-b** TUNEL (red) staining of control (**a**) and *tj>Dsor1 RNAi* (**b**) testes. **c** Number of TUNEL-positive cells in control (n=3) and *tj>Dsor1 RNAi* (n=3) testes. **d-e** Immunostaining of control (**d**) and *tj>Dsor1 RNAi* (**e**) testes for PH3 (red) and FasIII (green). **f** Number of PH3-positive cells in control (n=3) and *tj>Dsor1 RNAi* (n=3) testes. The W^1118^ line was used as control. Student’s *t*-test was used for the statistical analysis. **P < 0.01. Scale bar: 20 μm.

**Fig. S3 Somatic rl is required for GSC differentiation in *Drosophila* testes.** **a-b** Immunostaining of Zfh1 (red) and Eya (green) in control (**a**) and *tj>rl RNAi* (**b**) testes. **c** Number of Zfh1-positive cells in control (n=3) and *tj>rl RNAi* (n=3) testes. **d** Number of Eya-positive cells in control (n=3) and *tj>rl RNAi* (n=3) testes. **e-f** Immunostaining of Vasa (red) and 1B1 (green) in control (**e**) and *tj>rl RNAi* (**f**) testes. **g** Number of pointed fusomes in control (n=3) and *tj>rl RNAi* (n=3) testes. The W^1118^ line was used as control. Student’s *t*-test was used for the statistical analysis. **P* < 0.05, ****P* < 0.001, n.s. not significant. Scale bar: 20 μm.

**Fig. S4 Knockdown of *rl* in CySCs leads to the increase of cell death and proliferation. a-b** TUNEL (red) staining of control (**a**) and *tj>rl RNAi* (**b**) testes. **c** Nmber of TUNEL positive cells in control (n=3) and *tj>rl RNAi* (n=3) testes. **d-e** Immunostaining of control (**d**) and *tj>rl RNAi* (**e**) testes using PH3 (red) and FasIII (green). **f** Number of PH3 positive cells in control (n=3) and *tj>rl RNAi* (n=3) testes. The W^1118^ line was used as control. Student’s *t*-test was used for the statistical analysis. *P<0.05, ***P<0.001. Scale bar: 20 μm.

**Fig. S5 rl controls proliferation and cell survival in S2 cells.** **a** Relative mRNA level of *rl* in NC, *sirl-51* and *sirl-785* treated S2 cells. **b** Immunostaining of NC and *sirl-785­*-treated S2 cells using PH3 (red). **c** Percentages of PH3 positive cells in NC (n=3) and *sirl-785*-treated (n=3) S2 cells. **d** CCK-8 test in NC and *sirl-785*-treated S2 cells. **e** TUNEL (red) staining in NC and *sirl-785*-treated S2 cells. **f** Percentages of TUNEL positive cells in NC (n=3) and *sirl-785*-treated (n=3) S2 cells. **g** Cell components in NC and *sirl-785*-treated S2 cells by flow cytometry. **h** Percentages of cell components in NC (n=3) and *sirl-785*-treated (n=3) S2 cells. Student’s *t*-test was used for the statistical analysis. **P<0.01, ***P<0.001, n.s. not significant. Scale bar: 30 μm.

**Fig. S6 Inactivation of somatic rl results in aberrant dpERK expression.** **a-c** Immunostaining of control (**a**) and *tj>rl RNAi* (**b-c**) testes for Vasa (red) and dpERK (green). Panel (**c**) shows enlarged views of sections marked in (**b**). **d** Percentages of testes with germline dpERK signals in control (n=20) and *tj>rl RNAi* (n=9) testes. Chi-square test was used to evaluate for statistical differences of testes with germline dpERK signals. **e** Number of germline dpERK signals in control (n=10) and *tj>rl RNAi* (n=9) testes. Student’s *t*-test was used for statistical differences of the number of germline dpERK signals. ***P*<0.01, ***P<0.001. The W^1118^ line was used as control. Scale bar: 20 μm.
