# Supplementary figures and images for: Somatic CG6015 mediates cyst stem cell maintenance and germline stem cell differentiation via EGFR signaling in Drosophila testes
Source: Cell Death Discov. 2021 Apr 6;7:68. doi: 10.1038/s41420-021-00452-w (PMC8024382; doi:10.1038/s41420-021-00452-w)

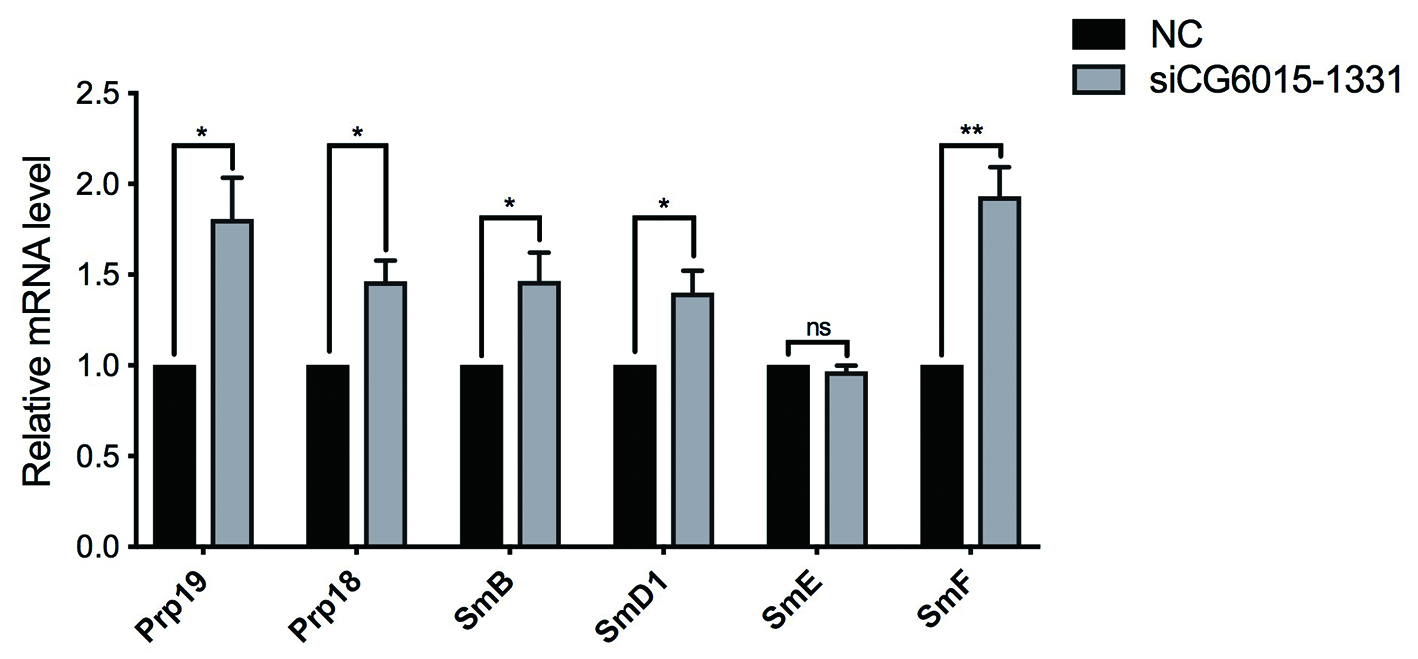

Supplement: Supplementary file 2 — Supplementary Figure S1 [file 41420_2021_452_MOESM2_ESM.tif]

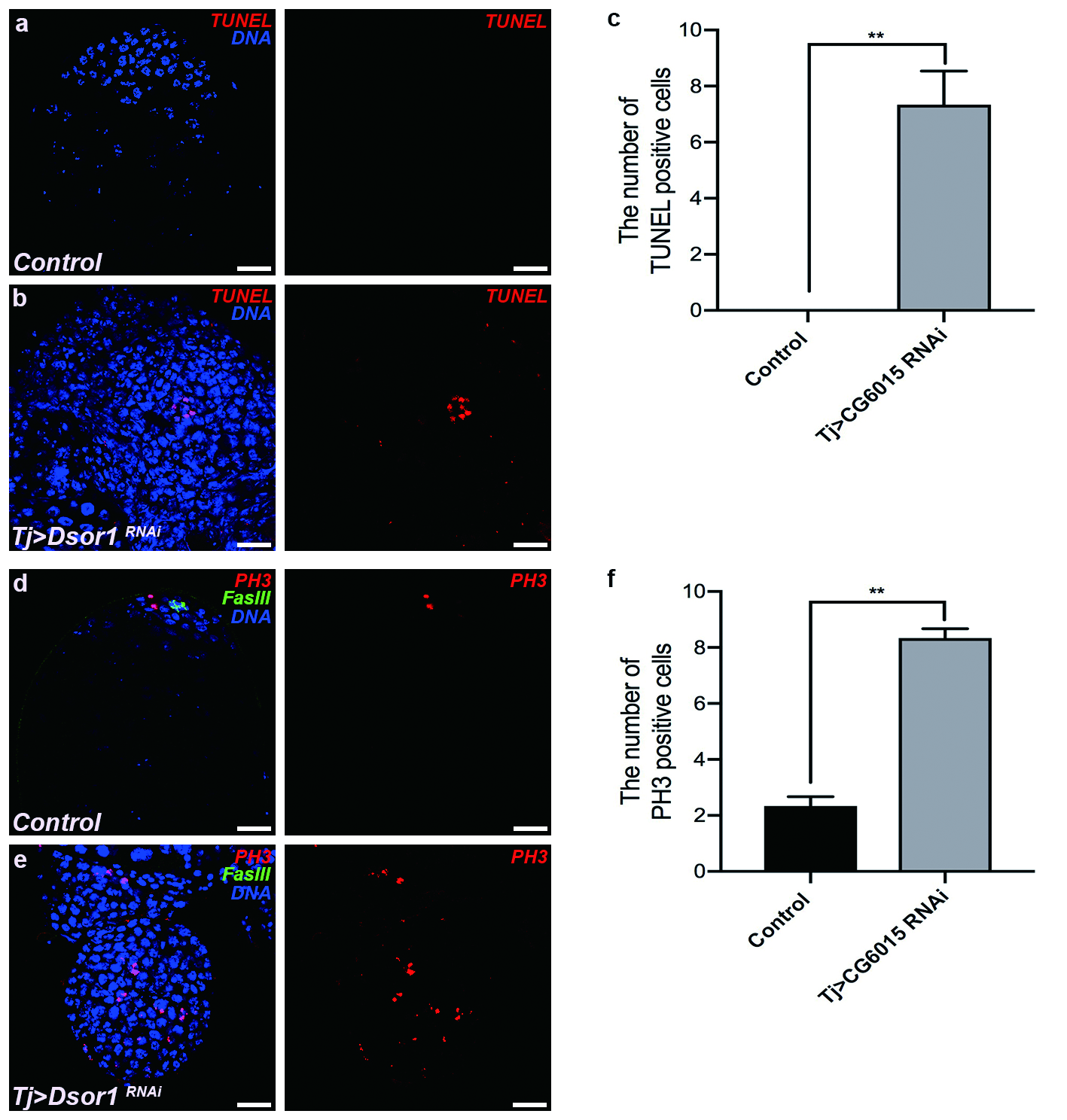

Supplement: Supplementary file 3 — Supplementary Figure S2 [file 41420_2021_452_MOESM3_ESM.tif]

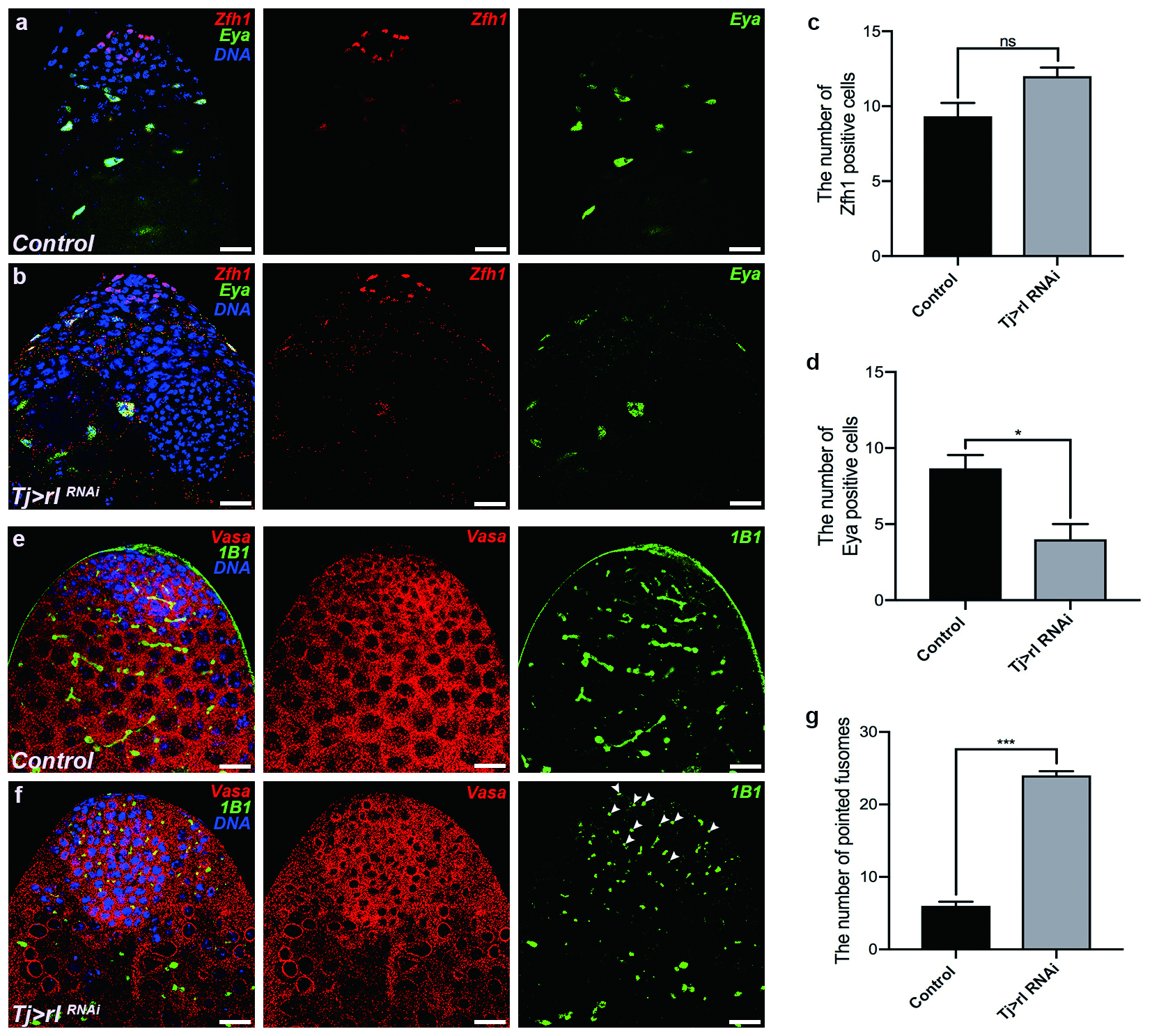

Supplement: Supplementary file 4 — Supplementary Figure S3 [file 41420_2021_452_MOESM4_ESM.tif]

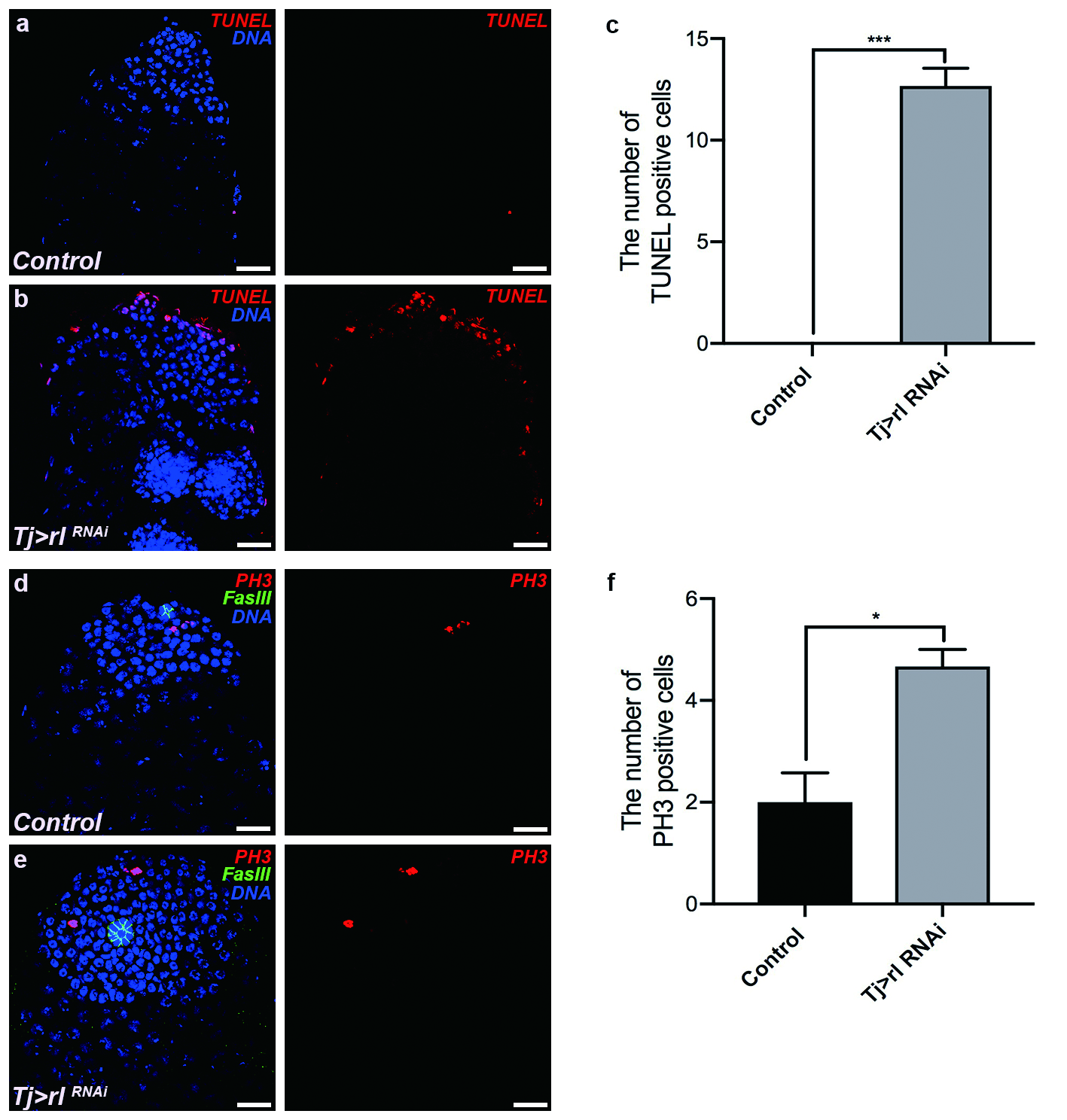

Supplement: Supplementary file 5 — Supplementary Figure S4 [file 41420_2021_452_MOESM5_ESM.tif]

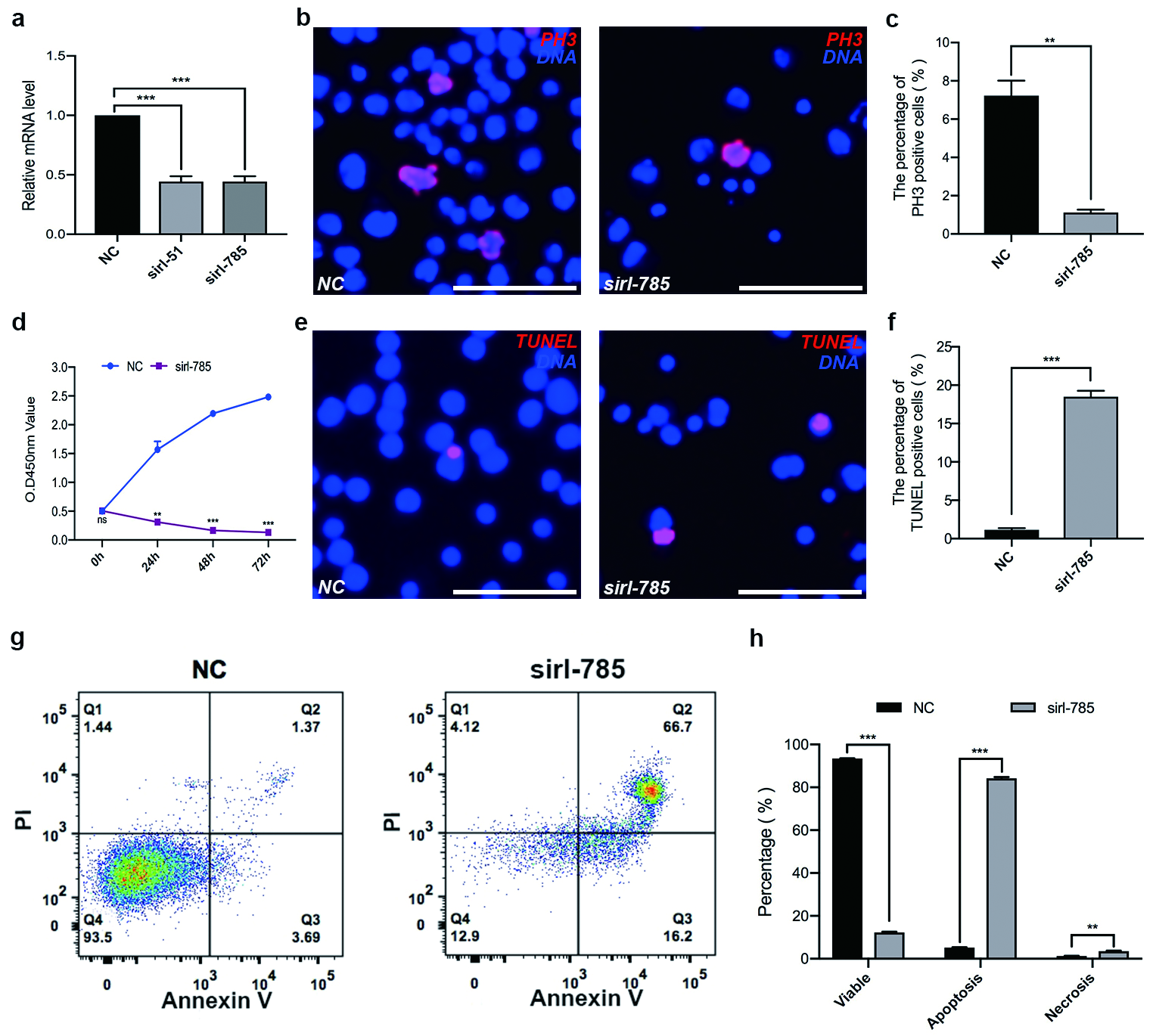

Supplement: Supplementary file 6 — Supplementary Figure S5 [file 41420_2021_452_MOESM6_ESM.tif]

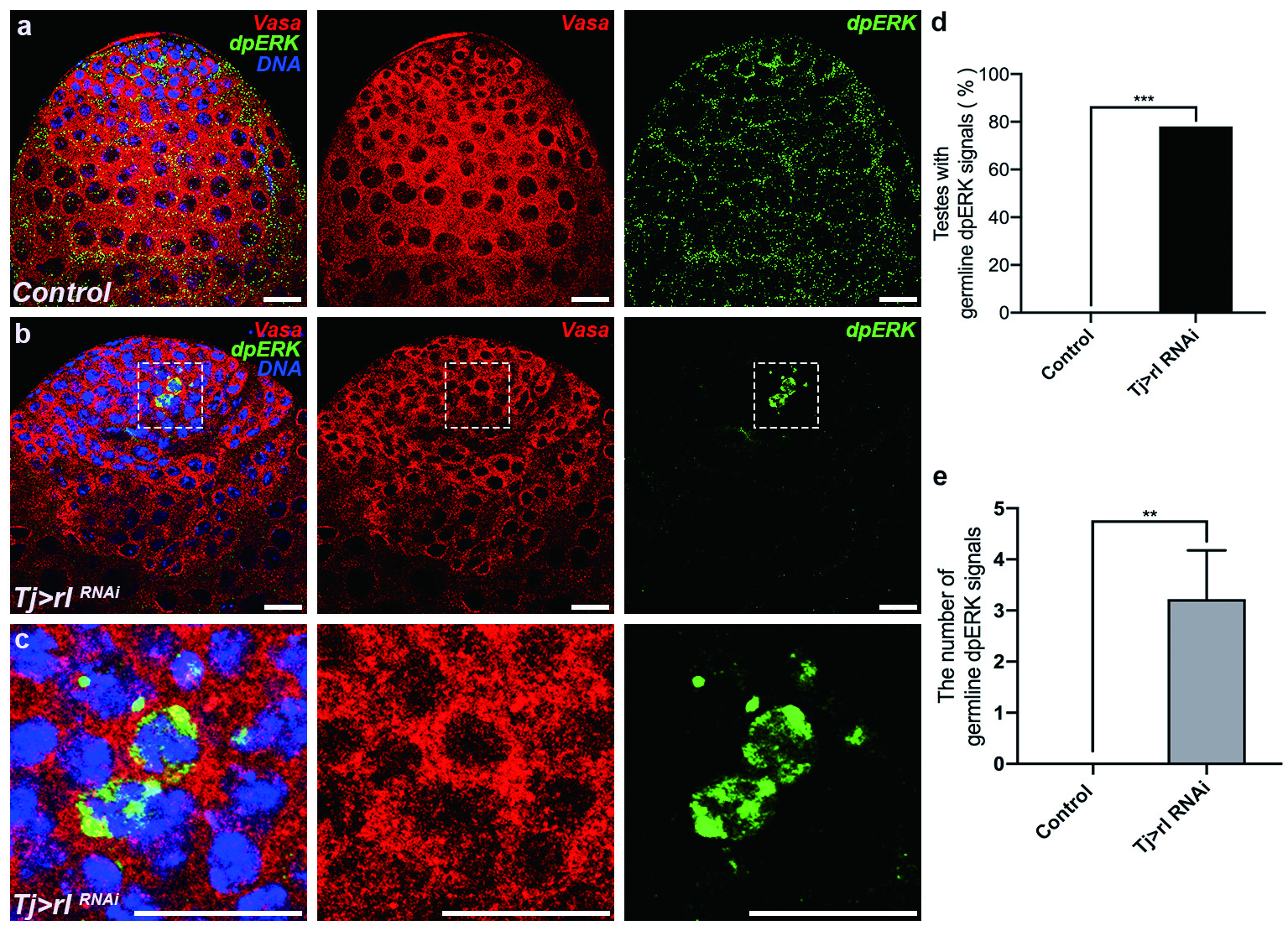

Supplement: Supplementary file 7 — Supplementary Figure S6 [file 41420_2021_452_MOESM7_ESM.tif]
