# Supplementary material for: Somatic CG6015 mediates cyst stem cell maintenance and germline stem cell differentiation via EGFR signaling in Drosophila testes
Source: Cell Death Discov. 2021 Apr 6;7:68. doi: 10.1038/s41420-021-00452-w (PMC8024382; doi:10.1038/s41420-021-00452-w)
Supplement: Supplementary file 8 — Supplementary Table S1 [file 41420_2021_452_MOESM8_ESM.docx]

**Table S1. Detailed information of siRNA used in this study.**

| **siRNA Name** | **Sense (5’-3’)** | **Antisense (5’-3’)** |
| --- | --- | --- |
| NC | UUCUCCGAACGUGUCACGUTT | ACGUGACACGUUCGGAGAATT |
| siCG6015-741 | GGGCCGCUCUUAUUUGCAUTT | AUGCAAAUAAGAGCGGCCCTT |
| siCG6015-1331 | GGGACAUUCCUGUGGACAUTT | AUGUCCACAGGAAUGUCCCTT |
| siDsor1-599 | GCGACAAUCACGCCAUCAUTT | AUGAUGGCGUGAUUGUCGCTT |
| siDsor1-686 | GCGUCUCCGGUCAACUGAUTT | AUCAGUUGACCGGAGACGCTT |
| sirl-51 | GGAAGUUCCUCAAUCUAAUTT | AUUAGAUUGAGGAACUUCCTT |
| sirl-785 | GGGACGAUUUAGAGUGUAUTT | AUACACUCUAAAUCGUCCCTT |
