# Supplementary material for: Somatic CG6015 mediates cyst stem cell maintenance and germline stem cell differentiation via EGFR signaling in Drosophila testes
Source: Cell Death Discov. 2021 Apr 6;7:68. doi: 10.1038/s41420-021-00452-w (PMC8024382; doi:10.1038/s41420-021-00452-w)
Supplement: Supplementary file 9 — Supplementary Table S2 [file 41420_2021_452_MOESM9_ESM.docx]

**Table S2. All primer sequences used in this study.**

| **Gene** | **Forward (5'-3')** | **Reverse (5'-3')** |
| --- | --- | --- |
| GAPDH | GTGGTGAACGGCCAGAAGAT | GCCTTGTCAATGGTGGTGAA |
| CG6015  Dsor1  rl | GCCTGCCAGTCGTTAGACAA  ACGCTGGAGGGTCTGGACATG  TGATCCTGGAGATGAGCCTGTCG | GCGTAGCCAGAGACCATGTG  TTCATCACCACGCCGCCATTG  TAAGGCGCATTGTCTGGTTGTCG |
| Prp19 | GCTGCCACGAAGGACCTGTTAC | CCTGTGCGGATATCGGAGAATGC |
| Prp18 | GCTGCTCACCTTCCTGCTCAAG | AGCGGCTTAACGTATTCCTTAGTCTG |
| SmB | CATGAACTTGATCCTCGGCGACTG | CCTCTGGCGGCGGTGGTC |
| SmD1 | CACCTGAAGAGCGTTCGGATGAC | TGTCGTCGATGAGGAGCGTCTC |
| SmE | CCATCAACCTGATCTTCCGTTACCTG | GCGTCGTCCAGCACCAGATTC |
| SmF | GCTCCGTGACTGGTAATCTTGGC | TCCTCGTCGTCGTCCTCCATG |
